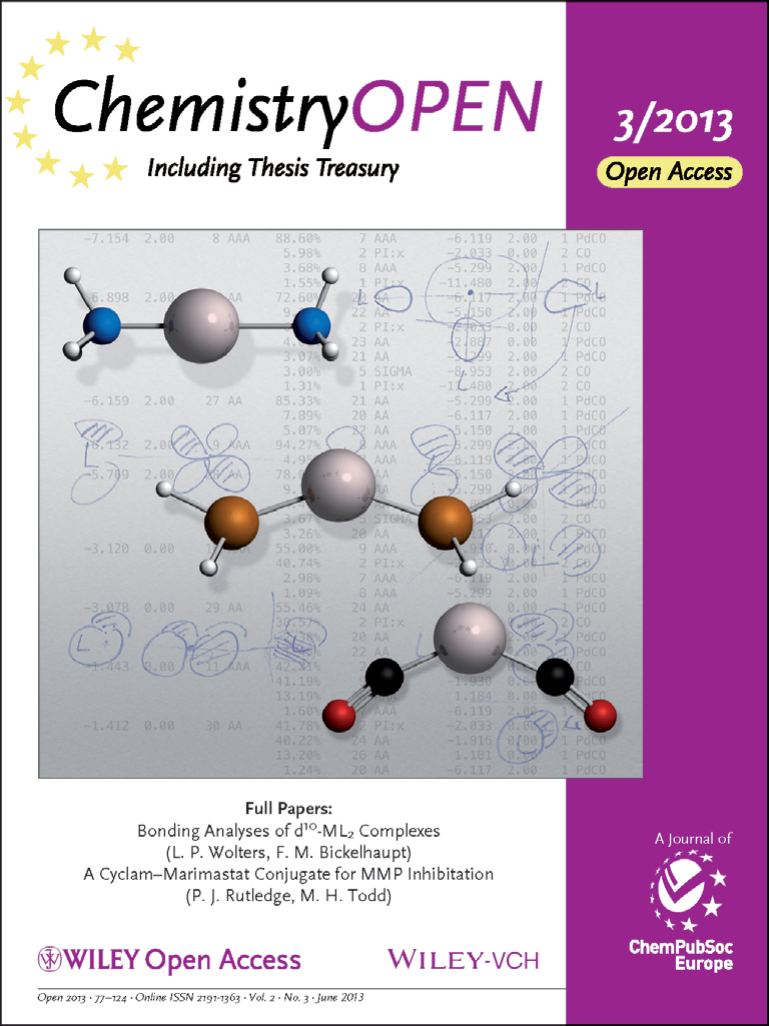# Nonlinear d^10^-ML_2_ Transition-Metal Complexes

**DOI:** 10.1002/open.201300026

**Published:** 2013-06-20

## Abstract

Invited for this month′s cover is the group of Prof. F. Matthias Bickelhaupt. The cover picture illustrates the authors′ quantum chemical finding that π electrons can significantly bend otherwise linear d^10^-ML_2_ complexes through backbonding. For more details, see the Full Paper on p. 106 ff.


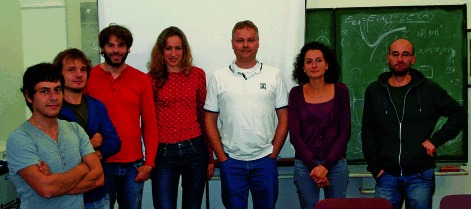


The Group of F. Matthias Bickelhaupt


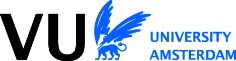


Department of Theoretical Chemistry Amsterdam Center for Multiscale Modeling VU University E-mail: F.M.Bickelhaupt@vu.nl


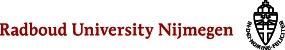


Institute for Molecules and Materials Radboud University Nijmegen

## What aspects of this project do you find most exciting?

The most exciting aspect of this project is, for me, that the initially, seemingly chaotic behavior of ML_2_ complexes (adopting in some cases the well-known linear arrangement and in other cases an “anomalous” nonlinear geometry) can eventually be understood in terms of a transparent molecular orbital (MO) picture that combines quantitative accuracy with the predictive power of a qualitative physical model.

## What future opportunities do you see (in the light of the results presented in this paper)?

The present result of a predictive MO model for the geometry of ML_2_ complexes provides new methods of tuning the bite angle. Thus, the activity of such complexes in catalytic processes can be controlled, namely through the intrinsic preference of the [ML_2_] moiety for adopting a particular bite angle instead of enforcing a bite angle in a chelating ligand “externally” using the molecular scaffold.

## Is your current research mainly curiosity driven (fundamental) or rather applied?

My current research is indeed mainly curiosity driven: I wish to obtain an understanding of the material world at the molecular level. Being able to compute what molecules do is nice. But a more fundamental understanding *why* they do so—for example, why they adopt the geometry they have or why they possess a particular reactivity—constitutes the true challenge and the ultimate objective of my profession. Because in this way, theory can contribute to a more rational design of new, valuable compounds, materials and processes which is chemistry′s core business.

## What other topics are you working on at the moment?

I am interested in developing novel chemical theories and methods for rationally designing molecules, nanostructures and materials as well as chemical processes toward these compounds, based on quantum mechanics and advanced computer simulations. My group is working in four main directions of research that are intimately connected and reinforce each other: (1) structure and chemical bonding in Kohn–Sham density functional theory, (2) supramolecular chemistry and theoretical biochemistry, (3) elementary chemical reactivity, and (4) fragment-oriented rational design of catalysts. Recently, projects have been launched in the fields of astrochemistry and solar cells.